# Complete Genome Sequence of *Mycoplasma cynos* Strain C142

**DOI:** 10.1128/genomeA.00196-12

**Published:** 2013-02-14

**Authors:** Caray A. Walker, Sally A. Mannering, Shelly Shields, Damer P. Blake, Joe Brownlie

**Affiliations:** a Department of Pathology and Infectious Diseases, the Royal Veterinary College, North Mymms, Hatfield, Hertfordshire, United Kingdom; b Veterinary Medicine Research & Development, Pfizer Inc., Kalamazoo, Michigan, USA

## Abstract

Here we report the *de novo* genome sequencing of *Mycoplasma cynos* strain C142, isolated from a dog with canine infectious respiratory disease (CIRD) in the United States.

## GENOME ANNOUNCEMENT

*Mycoplasma cynos* is a member of the class *Mollicutes*, a diverse group of cell wall-less bacteria which occupy a wide host range. Mycoplasmas are known commensals of the canine upper respiratory tract (1), although they have also been associated with respiratory disease. Several *Mycoplasma* species, including *M. canis*, *M. cynos*, and *M. edwardii*, have been isolated from the lower respiratory tracts of dogs with clinical disease (2–5). Canine infectious respiratory disease (CIRD) is a multifactorial disease involving both viral and bacterial agents. Symptoms of CIRD include a dry cough (often called kennel cough), anorexia, vomiting, and depression. Outbreaks of CIRD are particularly common in kennels where dogs are housed in close proximity to one another and where there is a constant flow of naive animals entering the group. Rehoming centers and training facilities often suffer endemic CIRD which is difficult to control. Recent evidence suggests that *M. cynos* in particular is an important etiological agent of CIRD (5–8). To date very little is known about the role *M. cynos* plays in CIRD, whether the bacterium is a primary pathogen or an opportunistic secondary pathogen. The lack of a genome sequence has limited molecular characterization of *M. cynos* and its role in CIRD pathogenesis.

*Mycoplasma cynos* C142 was isolated from the tracheal wash of a dog in the United States. The dog was exhibiting signs of respiratory disease, including a dry hacking cough and nasal discharge. The C142 isolate was considered a pure culture of *M. cynos*, and identity was confirmed by previously described detection methods, 16S sequencing, denaturing gradient gel electrophoresis (DGGE), and PCR (5, 9). Genomic DNA was extracted from *M. cynos* C142 following 4 to 5 passages on ME medium (Mycoplasma Experience Ltd., United Kingdom). The C142 genome was sequenced by using a combination of Illumina and 454 GS-FLX sequencing technologies, resulting in 73× genome coverage. The *de novo* genome assembly was completed by using the MIRA 3.1 assembly system. Approximately 248 Sanger sequencing reactions were carried out to close gaps and allow sequences to be assembled into nine contigs and ordered into one scaffold. The Prokov Hidden Markov model (HMM) was used to predict coding DNA sequences (CDS) (10). Following detection all CDS were translated into amino acid sequences and compared by BLASTP to public databases. Ribosomal RNA was detected using Meta-RNA (11). The complete *M. cynos* C142 genome is comprised of 998,123 bp in a single circular chromosome, with an average G+C content of 26% (comparable to *Mycoplasma mycoides* at 24%, lower than *Mycoplasma pneumoniae* at 40%) (12). A total of 883 CDS were predicted, resulting in approximately 1 CDS per kilobase, 491 of which have been assigned a putative functional identity, including 240 within the Kyoto Encyclopedia of Genes and Genomes (KEGG) metabolism pathway, 136 within genetic information processing, 41 within environmental information processing, and 5 within cellular processes. The sequence described here provides a valuable new resource for the study of CIRD.

### Nucleotide sequence accession number.

The genome sequence of the *M. cynos* strain C142 has been deposited in the EMBL nucleotide sequence database under accession number HF559394.
